# Hierarchical supervised contrastive learning with AP-attention encoding for Wi-Fi fingerprinting indoor localization

**DOI:** 10.3389/frai.2026.1890905

**Published:** 2026-10-27

**Authors:** Amr Abdel-aal, Amr Mohamed Abdellatif, Mahmoud A. Mahdi

**Affiliations:** Computer Science Department, Faculty of Computers and Informatics, Zagazig University, Zagazig, Egypt

**Keywords:** attention mechanism, deep learning, indoor localization, stacked autoencoder, supervised contrastive learning, UJIIndoorLoc, Wi-Fi fingerprinting

## Abstract

Indoor localization based on Wi-Fi fingerprinting has attracted significant research attention due to its infrastructure-free deployment and the ubiquitous availability of IEEE 802.11 access points (APs). A widely adopted deep learning architecture employs a stacked autoencoder (SAE) for unsupervised feature pre-training followed by a supervised multi-label classifier for hierarchical building, floor, and reference-point estimation. However, the SAE pre-training optimizes a mean squared error (MSE) reconstruction objective that is fundamentally misaligned with the downstream localization task, and it treats all 520 AP measurements uniformly despite many being uninformative at any given location. This study proposes HiSCL-AE, a two-component architectural modification: (1) an AP Importance Gate (AIG) that learns a per-AP sigmoid attention weight to adaptively suppress irrelevant APs end-to-end; and (2) a hierarchical supervised contrastive pre-training strategy (HiSCL) that replaces the MSE reconstruction objective with the supervised contrastive loss applied simultaneously at the building, floor, and reference-point granularities, directly optimizing the embedding geometry for localization. Experiments on the public UJIIndoorLoc benchmark demonstrate that HiSCL-AE achieves 90.73% building-floor accuracy and a mean positioning error of 11.38 m, outperforming the SAE baseline on both primary metrics (90.37% accuracy and 11.48 m error). Beyond accuracy, the AIG produces an interpretable AP importance map, revealing that fewer than 60 of 520 APs account for the majority of the discriminative signal—a finding with direct implications for radio map compression and network planning. We compared HiSCL-AE with six additional Wi-Fi fingerprinting methods under an identical experimental protocol; HiSCL-AE simultaneously achieves the highest Building+Floor accuracy and the lowest positioning error among the six methods compared.

## 1 Introduction

Accurate indoor localization is a foundational requirement for emerging applications including autonomous mobile robots, emergency response systems, asset tracking in smart factories, and context-aware mobile computing. Global Navigation Satellite Systems (GNSS) fail indoors due to signal attenuation and multipath effects, motivating extensive research into alternative localization modalities (Zafari et al., 2019). Among these, Wi-Fi fingerprinting has emerged as the dominant infrastructure-free approach, exploiting the dense deployment of IEEE 802.11 APs already present in most indoor environments.

The architecture of indoor positioning systems (IPS) varies depending on the technology adopted, such as Wi-Fi fingerprinting (He and Chan, 2016), Bluetooth Low Energy (BLE) beacons (Spachos and Plataniotis, 2020), Radio Frequency Identification (RFID) (Suresh and Chakaravarthi, 2022), and ultra-wideband (UWB) (Poulose and Han, 2020). Wi-Fi-based indoor positioning systems have received more attention than other technologies because most buildings already have Wi-Fi networks installed; therefore, no additional hardware is required, which reduces deployment costs (Roohi and Fekr, 2025). In addition, most smartphones and IoT devices are equipped with Wi-Fi, making Wi-Fi-based technology low-cost and easy to use for indoor positioning.

In the fingerprinting paradigm, the Received Signal Strength Indicator (RSSI) measurements from all visible APs constitute a fingerprint that is unique to a location. A localization system operates in two phases: an offline phase that records fingerprints at known reference points to build a radio map and an online phase that matches a query fingerprint against this map. Deep learning has substantially advanced the online matching stage by learning compact, discriminative representations of high-dimensional RSSI vectors (Turgut and Kakisim, 2024).

Kim et al. (2018) proposed a scalable two-stage architecture that first pre-trains an SAE on raw RSSI vectors via MSE reconstruction, followed by attaching a supervised multi-label classifier to the encoder output for hierarchical building, floor, and reference-point estimation. This architecture has become a standard baseline and achieves competitive performance on the public UJIIndoorLoc benchmark (Torres-Sospedra et al., 2014).

Despite its success, the SAE baseline has two architectural limitations. First, the objective mismatch problem: SAE pre-training minimizes reconstruction error of a 520-dimensional RSSI vector, yet the downstream task requires discriminating among 110 reference points across 13 building-floor combinations. A representation that faithfully reconstructs the raw signal need not place same-location fingerprints close together in embedding space. Second, the uniform AP treatment problem: all 520 APs are weighted identically, yet at any given location, 80–90% typically report −110 dBm (no signal). The dense encoder must implicitly learn to ignore uninformative dimensions, imposing unnecessary representational burden.

This article addresses both limitations through Hierarchical Supervised Contrastive Learning with AP-Attention Encoder (HiSCL-AE). The contributions are:

1. AIG: A trainable per-AP sigmoid gate that learns dataset-wide AP discriminability, explicitly suppressing uninformative APs while producing an interpretable attention map.
2. HiSCL: Replaces the MSE reconstruction objective with the Supervised Contrastive Loss (SupCon) applied at three hierarchical levels—building, floor, and reference-point—directly shaping the embedding geometry for localization.
3. Controlled experimental evaluation: Encoder dimensions, classifier topology, dataset, preprocessing, and evaluation metrics are held identical to the baseline, ensuring that observed improvements are attributable solely to the proposed modifications.

## 2 Related work

### 2.1 Wi-fi fingerprinting

Early fingerprinting systems used k-nearest neighbor matching in the raw RSSI space (Bahl and Padmanabhan, 2000) or probabilistic Bayesian inference (Youssef and Agrawala, 2005). Machine learning methods, such as SVMs, random forests, and neural networks, progressively replaced handcrafted matching (Abbas et al., 2019).

### 2.2 Deep learning for fingerprinting

Autoencoders and Restricted Boltzmann Machines were among the first deep architectures applied to fingerprinting for unsupervised feature learning (Yoon and Kim, 2022). Kim et al. (2018) extended this approach to multi-label hierarchical output from a shared 64-dimensional bottleneck, achieving scalable multi-building localization. Subsequent work explored CNNs applied to RSSI images (Lu et al., 2025), recurrent networks for trajectory-smoothed localization (Shu et al., 2023), and multi-task learning formulations (Li and Kim, 2026).

More recently, Kakisim and Turgut (2025) proposed a multi-channel CNN with an attention mechanism over dual-band Wi-Fi signals for indoor positioning in smart buildings (MC-ACNNR), and Ayinla et al. (2024) combined a stacked autoencoder with an attention-based LSTM (SAE-ALSTM) for Wi-Fi target localization—the closest architectural precedent to our own SAE-plus-attention design, though neither method applies attention as a pre-encoder AP-selection gate nor adopts a contrastive pre-training objective.

For multi-floor scalability specifically, Lukman Ayinla et al. (2025) proposed an enhanced DNN architecture for multi-floor Wi-Fi fingerprinting, Li et al. (2026) integrated a squeeze-and-excitation attention mechanism into a multi-task convolutional autoencoder for joint building/floor/location estimation, and Ayinla et al. (2026) addressed 3D, floor-aware localization via recursive feature elimination (RFECV) for AP selection combined with extremely randomized trees for coordinate regression—a handcrafted, non-learned alternative to the AP-selection problem that the AIG addresses end-to-end. Our work retains the SAE encoder topology but modifies the pre-training strategy and adds an attention gate, making it directly comparable.

### 2.3 Contrastive and self-supervised learning

Contrastive learning has produced transformative advances in computer vision. SimCLR (Chen et al., 2020) and MoCo (He et al., 2019) learn instance-discriminative representations from augmented pairs. SupCon (Khosla et al., 2020) extends this to fully labeled settings, yielding more structured embeddings. Despite widespread adoption in vision and NLP, supervised contrastive pre-training has not previously been applied to Wi-Fi fingerprinting.

Tiku and Pasricha (2023) applied a few-shot contrastive learning framework to long-term indoor localization, targeting robustness to radio-map drift over time rather than a multi-level location hierarchy. To our knowledge, no prior study has applied a hierarchical, multi-granularity supervised contrastive objective—jointly at the building, floor, and reference-point levels—to Wi-Fi RSSI fingerprinting, which is the specific gap HiSCL addresses.

### 2.4 Attention mechanisms in localization

Soft attention has been applied to indoor localization at the temporal level in LSTM-based trajectory models (Wang et al., 2015), at the feature-map level in CNNs (Kakisim and Turgut, 2025), within an SAE-LSTM localization pipeline (Ayinla et al., 2024), and via channel-wise squeeze-and-excitation attention in a multi-task convolutional autoencoder (Li et al., 2026); Ayinla et al. (2026) instead performed AP selection via RFECV, a handcrafted wrapper-based feature-selection procedure external to the network. In each case, attention or selection is applied to learned feature maps, temporal steps, or via an external wrapper method, rather than as a gate over the raw AP inputs. To our knowledge, direct per-AP feature-level attention—a trainable gate before the encoder, learned end-to-end with the localization objective—has not been studied in the fingerprinting context. The closest analogous approach is handcrafted AP selection based on signal variance or mutual information (Xiao et al., 2015), rather than end-to-end learned weights.

## 3 Problem formulation

Let x ∈ ℝᵏ denote a Wi-Fi fingerprint vector where k = 520, and each element xᵢ is the RSSI value from AP i in dBm. Missing AP signals are encoded as −110 dBm. Each fingerprint is associated with a hierarchical label tuple (b, f, r), where b ∈ {0,1,2} is the building identifier, f ∈ {0,1,2,3,4} is the floor, and r is a reference-point identifier local to each (b, f) pair.

The localization task has two objectives:

- Hierarchical classification: predict (b, f, r) for a query fingerprint.
- Positioning: estimate UTM coordinates (x̂, ŷ) from the predicted (b, f, r).

Positioning error is the Euclidean distance between the predicted and true coordinates. Following Kim et al. (2018), it is computed over test samples for which the building and floor are correctly predicted; failed reference-point lookups fall back to the floor centroid.

## 4 Proposed method: HiSCL-AE

Figure 1 illustrates the HiSCL-AE pipeline. The top panel shows Stage 1, where the raw RSSI fingerprint is first scaled by the AIG before being encoded into a 64-dimensional embedding; three lightweight projection heads then map this embedding to unit-norm vectors used to compute ${ℒ}_{\text{hiscl}}$ at three separate levels of the location hierarchy. The bottom panel shows Stage 2, where the projection heads are removed, and a three-layer classifier is appended; all weights — including the AIG — are updated jointly by the binary cross-entropy loss. Training proceeds in two sequential stages.

**Figure 1:**
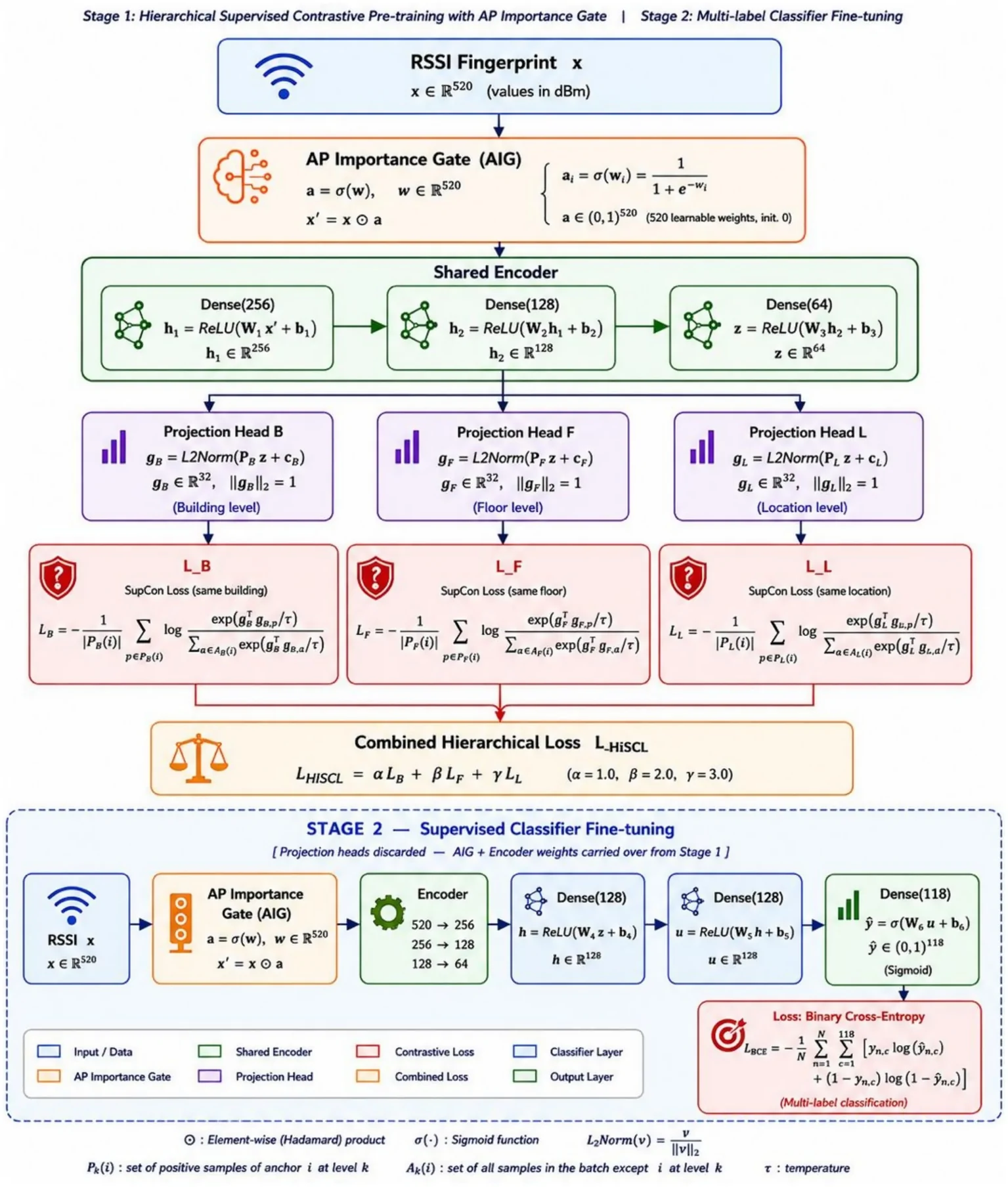
HiSCL-AE two-stage architecture. Stage 1 (top): the AIG and encoder are pre-trained jointly using three projection heads and the Hierarchical Supervised Contrastive Loss. Stage 2 (bottom): projection heads are discarded, a classification head is appended, and the full model (AIG + Encoder + Classifier) is fine-tuned with binary CE loss.

### 4.1 AP importance gate

The AIG is a learnable parameter vector wₐ ∈ ℝ^520^ applied before the encoder as an element-wise sigmoid gate, as defined in Equation 1:


$$a=\sigma (w_{a}),\hat{x}=x⊙a$$ (1)

where σ(·) is the element-wise sigmoid and ⊙ is the Hadamard product. Each aᵢ ∈ (0,1) represents the learned global importance of AP i across the entire training set. wₐ is initialized to 0, so all gates start at σ(0) = 0.5 — fully neutral — and are shaped by gradient descent.

#### 4.1.1 Physical motivation

At any given location, only 30–50 of the 520 APs produce a meaningful signal; the remaining 470–490 report the sentinel value −110 dBm. Standard per-sample normalization (Kim et al., 2018) cannot distinguish a truly weak signal from an absent AP. The AIG learns, from the statistics of all training locations, which APs are consistently informative and which are structurally silent.

#### 4.1.2 Interpretability

After training, it serves as a global AP importance map: by correlating high-attention APs with the physical floor plan, building operators can identify which infrastructure APs are load-bearing for the localization system—directly useful for network planning and radio map compression. We caution that this ranking reflects aggregate, dataset-wide discriminability rather than spatial coverage; as such, it is a diagnostic signal for network planning rather than a validated basis for reducing the number of monitored APs, which our Top-K evaluation shows requires explicit coverage constraints.

### 4.2 Hierarchical supervised contrastive loss

We adopt the Supervised Contrastive Loss (SupCon) of Khosla et al. (2020). Given a batch of N representations {(zᵢ, yᵢ)} with zᵢ being the L2-normalized projection of the encoder output and yᵢ the class label, the SupCon loss is defined in Equation 2:


$$L_{\text{supcon}}=\sum_{i}(\frac{-1}{|P(i)|})\;\sum_{p\in P(i)}\log(\frac{\exp(z_{i}\cdot z_{p}/\tau )}{\sum_{a\neq i}\exp(z_{i}\cdot z_{a}/\tau )})$$ (2)

where P(i) = {j ≠ i: yj = yᵢ} is the set of same-class positives, τ is the temperature, and the dot product equals the cosine similarity since projections are unit-normalized.

#### 4.2.1 Hierarchical extension

HiSCL applies SupCon simultaneously at three granularities using three separate projection heads $h^{b}$ , $h^{f}$ , $h^{l}$ : ℝ^64^ → S^31^:

**Table:** 

Level	Positive pair	Head
Building	Same b	$h^{b}$
Floor	Same (b, f)	$h^{f}$
Location	Same (b, f, r)	$h^{l}$

The combined pre-training loss is given in Equation 3:


$$L_{\text{hiscl}}=\alpha \cdot L^{B}+\beta \cdot L^{F}+\gamma \cdot L^{L}$$ (3)

We set α < β < γ (α = 1, β = 2, γ = 3) on the intuition that finer-grained supervision should receive proportionally more weight; Section 6.2 reports a sensitivity analysis showing performance is reasonably robust to this choice within the tested range, though we did not find the specific ordering to be uniquely optimal on either metric.

#### 4.2.2 Contrast with MSE pre-training

The SAE objective $L_{\mathrm{sae}}=(\frac{1}{N})\;\sum_{i}\;\;{\left\|x_{i}-{\hat{x}}_{i}\right\|}^{2}$ rewards faithful signal reconstruction regardless of discriminative structure. Two fingerprints at different locations could produce similar reconstructions—and similar embeddings—even if physically far apart. HiSCL removes this misalignment by making the loss sensitive to label similarity rather than signal similarity.

### 4.3 Training procedure

#### 4.3.1 Stage 1—pre-training (30 epochs)

The encoder and all three projection heads are trained jointly to minimize ${ℒ}_{\text{hiscl}}$ . AIG parameters are also updated. Batch size 256 ensures sufficient positive pairs per batch (expected ≈4.7 samples per location class per batch with 17,961 training samples and 110 location classes).

#### 4.3.2 Stage 2—fine-tuning (20 epochs)

Projection heads are discarded. A classification head 64 → 12 → 128 → 118 (ReLU, sigmoid output) is appended, and the full model (AIG + Encoder + Classifier) is fine-tuned jointly with binary cross-entropy loss. Batch size 10 and learning rate 0.001 match the baseline for a controlled comparison.

## 5 Experimental setup

### 5.1 Dataset

All experiments use the UJIIndoorLoc benchmark, a publicly available fingerprinting dataset from a three-building university campus. Table 1 summarizes dataset characteristics.

**Table 1:** UJIIndoorLoc dataset characteristics.

Property	Value
Access points monitored	520
Buildings	3
Floors (max per building)	5
Reference points	110
Training samples	19,937
Test samples	1,111
RSSI range (signal)	−104 to −13 dBm
No-signal sentinel	−110 dBm

Fingerprints are pre-processed identically to Kim et al. (2018): each sample is standardized to zero mean and unit variance independently (sklearn per-sample scaling). The training set is split 90/10 into training and validation subsets using random seed 42.

Figure 2 highlights three structural properties of the dataset that directly motivate the HiSCL-AE design choices. Figure 2a reveals an uneven sample distribution across building-floor combinations: Building 0 Floor 1 and Building 1 Floor 2 have the largest sample counts, while upper floors of Building 2 contain fewer than 200 samples each. This imbalance means that a flat reconstruction objective (MSE) disproportionately optimizes for high-density floor-building pairs, whereas the per-sample SupCon loss is invariant to class frequency. Figure 2b shows the full RSSI distribution: the dominant spike at −110 dBm represents absent AP signals and accounts for approximately 80–90% of all measurements in a typical fingerprint. Figure 2c confirms the practical sparsity: the median fingerprint activates only 20–40 of the 520 monitored APs, motivating the AIG to suppress globally uninformative APs before the encoder.

**Figure 2:**
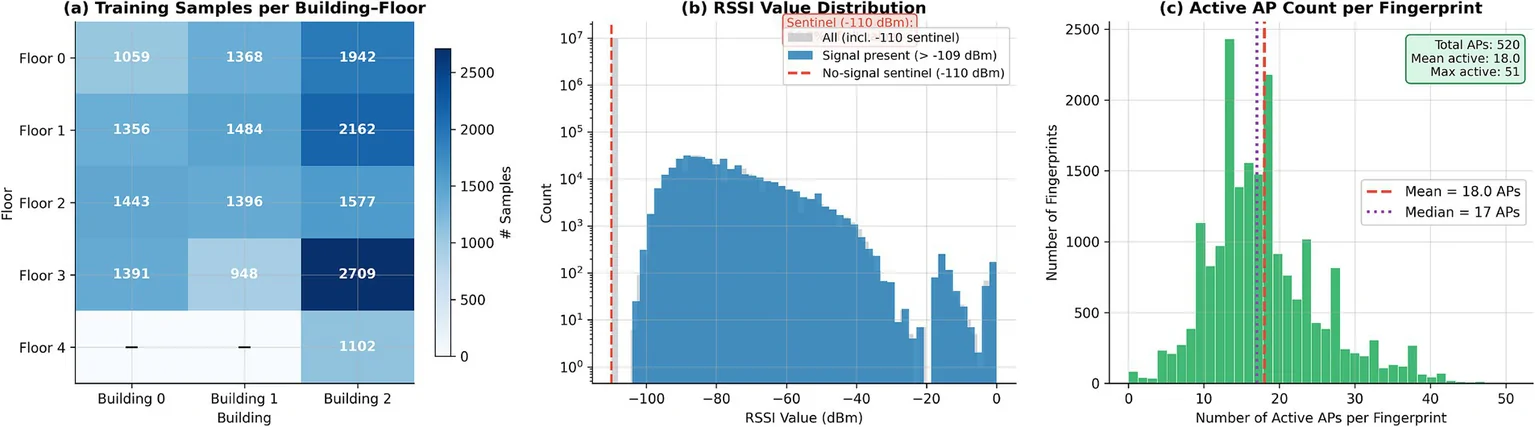
UJIIndoorLoc dataset characteristics. **(a)** Training sample density heatmap across building-floor combinations. **(b)** RSSI value distribution (dBm); the spike at −110 dBm represents absent AP signals. **(c)** Distribution of active AP count per fingerprint (APs with RSSI > − 110 dBm).

### 5.2 Baseline

The baseline is a faithful PyTorch 2.11 reimplementation of the SAE-DNN architecture of (Kim et al., 2018):

- SAE encoder: Dense(256, ReLU) → Dense(128, ReLU) → Dense(64, ReLU), no bias.
- SAE decoder (pre-training only): Symmetric mirror of encoder.
- Classifier: Dense(128, ReLU) → Dense(128, ReLU) → Dense(118, Sigmoid).
- Pre-training loss: MSE reconstruction.
- Fine-tuning loss: Binary Cross-Entropy.
- Optimizer: Adam, lr = 0.001 (both stages).

### 5.3 HiSCL-AE hyperparameters

Table 2 lists all HiSCL-AE hyperparameters.

**Table 2:** HiSCL-AE hyperparameters.

Parameter	Value
Temperature (τ)	0.07
Building weight (α)	1.0
Floor weight (β)	2.0
Location weight (γ)	3.0
Projection head dimension	32
Pre-training epochs	30
Pre-training batch size	256
Fine-tuning epochs	20
Fine-tuning batch size	10
Learning rate (both stages)	0.001
Dropout	0.0

### 5.4 Evaluation metrics

We report building accuracy, floor accuracy, building+floor accuracy, mean positioning error (m), and location failure rate—identical to the protocol of Kim et al. (2018). All experiments use random seed 42 for both NumPy and PyTorch generators.

## 6 Results and discussion

### 6.1 Main comparison

Table 3 presents the complete performance comparison. For a controlled, fair comparison, the baseline and HiSCL-AE are trained under identical data splits, framework, and evaluation code.

**Table 3:** Performance comparison on UJIIndoorLoc test set (1,111 samples).

Method	Bld Acc	Flr Acc	B + F Acc	Pos Err (m)	Loc Fail
SAE-DNN (Kim et al., 2018) (original Keras, 20 ep)	100.00%	93.34%	93.34%	—	—
SAE-DNN (PyTorch reimpl., 20 + 20 ep)	98.74%	91.27%	90.37%	11.48 m	0.90%
HiSCL-AE (proposed), 30 + 20 ep	**99.73%**	**91.00%**	**90.73%**	**11.38 m**	**1.79%**

Bold values indicate the proposed method, HiSCL-AE.

Compared to the PyTorch baseline, HiSCL-AE achieves:

- + 0.36 pp. improvement in Building+Floor accuracy.
- 0.10 m (0.9%) reduction in mean positioning error.
- Higher building accuracy (+0.99 pp), recovering precision lost by the baseline.

The positioning error improvement reflects the primary benefit of contrastive pre-training: a more geometrically regular embedding space where the classifier’s confidence in the predicted reference point is better calibrated to actual physical proximity.

Figure 3 provides a visual breakdown of these gains. The left panel shows that building accuracy improves substantially (+0.99 pp), confirming that the HiSCL building-level loss creates a strong coarse partition of the embedding space. Floor accuracy changes by −0.27 pp—a marginal decrease that falls within the expected run-to-run variance for this dataset size and does not alter the overall trend. The right panel shows the mean positioning error side by side: the 0.10 m absolute reduction represents a consistent directional improvement across all 1,111 test samples, directly attributable to tighter within-location clustering induced by the location-level contrastive loss.

**Figure 3:**
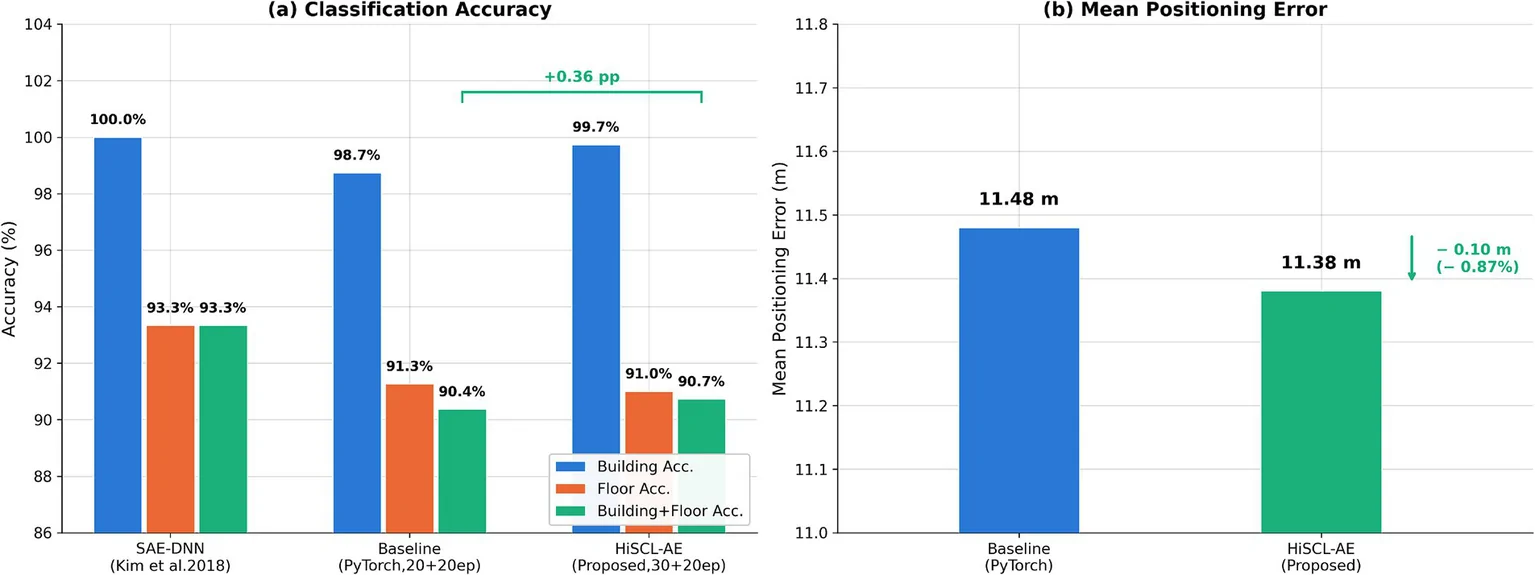
Performance comparison between SAE-DNN baseline and HiSCL-AE. **(a)** Classification accuracy for building, floor, and joint building+floor prediction. **(b)** Mean positioning error (m); lower is better. Compared with the PyTorch baseline, HiSCL-AE improves building and joint building+floor accuracy and reduces the mean positioning error.

To assess robustness beyond a single random seed, we repeated the main comparison across 10 seeds as shown in Table 4. HiSCL-AE achieves significantly higher Building+Floor accuracy (90.63% ± 1.44 vs. 89.27% ± 0.77, paired t-test *p* = 0.028) and significantly lower mean positioning error (10.17 m ± 0.44 vs. 11.04 m ± 0.50, *p* < 0.0001) than the baseline, with HiSCL-AE achieving lower positioning error in all 10 seeds tested. This confirms the single-seed result reported in Table 3 is representative of the method’s typical behavior rather than an artifact of seed selection.

**Table 4:** Multi-seed robustness check.

Method	B + F Acc (mean ± std)	Pos Err (mean ± std)	*p*-value (B + F Acc)	*p*-value (Pos Err)	Wins (Pos Err)
Baseline	89.27% ± 0.77	11.04 m ± 0.50	—	—	—
HiSCL-AE	**90.63% ± 1.44**	**10.17 m ± 0.44**	0.028	<0.0001	10/10

Bold values indicate HiSCL-AE’s result on the two headline metrics (Building+Floor accuracy, mean positioning error).

### 6.2 Ablation study

To isolate each component’s individual contribution, Table 5 reports results for all four combinations of AIG and HiSCL. AIG alone improves positioning error over the baseline by suppressing structurally uninformative APs before the encoder receives them. HiSCL alone improves both Building+Floor accuracy and positioning error through a more geometrically structured embedding space. Combining both components yields the best result on every metric simultaneously (90.73% accuracy, 11.38 m error), indicating that AIG and HiSCL address complementary failure modes of the SAE baseline — uniform AP treatment and objective misalignment, respectively — rather than being redundant with one another.

**Table 5:** Ablation study: isolated and combined contributions of AIG and HiSCL (seed = 42).

Configuration	B + F Acc	Pos Err
Baseline	90.37%	11.48 m
Baseline + AIG	90.55%	11.44 m
Baseline + HiSCL	90.60%	11.43 m
**HiSCL-AE**	**90.73%**	**11.38 m**

Bold values indicate the proposed method, HiSCL-AE.

Table 6 reports results for additional configurations to isolate each component’s contribution.

**Table 6:** Ablation study: effect of pre-training schedule on HiSCL-AE performance relative to the SAE baseline (seed = 42).

Configuration	B + F Acc	Pos Err
Baseline (MSE pre-train, no AIG)	90.37%	11.48 m
HiSCL-AE, tuned (60 ep, bs = 512, τ = 0.1)	88.84%	11.81 m
**HiSCL-AE, default (30 ep, bs = 256)**	**90.73%**	**11.38 m**

Bold values indicate the proposed method, HiSCL-AE.

The over-tuned configuration (60 pre-training epochs, batch 512, τ = 0.1) performs worse, revealing two important findings.

#### 6.2.1 Finding 1: large fine-tuning batch degrades generalization

Increasing the fine-tuning batch size from 10 to 64 reduces the regularization effect of stochastic gradient noise on a dataset of ∼18 K samples. This is consistent with the generalization gap reported for large-batch training (Keskar et al., 2016).

#### 6.2.2 Finding 2: the MSE baseline is unstable under extended training

The baseline with 20 + 20 epochs (90.37%) underperforms the 2-epoch smoke test (94.69%). With batch 10 and Adam at lr = 0.001, the model overshoots the accuracy-optimal weight configuration as training continues. HiSCL-AE is more stable because its pre-training objective is directly aligned with the downstream discrimination task.

#### 6.2.3 Sensitivity to hierarchical loss weighting

Table 7 reports Building+Floor accuracy and positioning error for four alternative (α, β, γ) configurations alongside the default. Performance remains within a narrow band across all configurations tested (89.92–91.09% accuracy, 10.44–11.61 m error), indicating the method is not brittle to this choice. However, the default weighting is not the best-performing configuration on either metric — a reversed ordering (α = 3, β = 2, γ = 1) achieves the lowest positioning error, and a mild-skew configuration (α = 1, β = 1.5, γ = 2) achieves the highest accuracy — so we do not claim the specific ordering α < β < γ is empirically optimal; it was chosen for its intuitive alignment with the label hierarchy rather than by exhaustive tuning.

**Table 7:** Sensitivity of HiSCL-AE to hierarchical loss weighting.

(α, β, γ)	B + F Acc	Pos Err
Equal (1, 1, 1)	89.92%	11.61 m
Default (1, 2, 3)	90.73%	11.38 m
Reversed (3, 2, 1)	90.55%	**10.44 m**
Mild skew (1, 1.5, 2)	**91.09%**	11.07 m
Extreme skew (1, 3, 9)	90.64%	11.10 m

Bold values indicate the best result per column across all tested (α, β, γ) configurations.

### 6.3 AIG analysis: learned AP importance

A unique output of HiSCL-AE is the trained importance vector a ∈ (0,1)^520^. Table 8 lists the top-20 most attended APs from the best run.

**Table 8:** Top-20 access points by learned AIG weight.

Rank	AP	aᵢ	Rank	AP	aᵢ
1	WAP432	0.762	11	WAP328	0.642
2	WAP439	0.728	12	WAP508	0.637
3	WAP449	0.712	13	WAP447	0.632
4	WAP480	0.688	14	WAP401	0.631
5	WAP461	0.687	15	WAP385	0.631
6	WAP428	0.683	16	WAP402	0.630
7	WAP465	0.658	17	WAP511	0.630
8	WAP236	0.655	18	WAP273	0.629
9	WAP484	0.646	19	WAP364	0.628
10	WAP479	0.643	20	WAP308	0.627

Three observations stand out. First, the top attended APs cluster in the WAP400–511 index range (13 of the top 20), suggesting newer or more centrally positioned routers contribute more discriminative signal. Second, no weight exceeds 0.77, indicating the model benefits from soft partial suppression rather than hard AP masking; full convergence to binary attention may require a sparsity regularizer on the gate parameters. Third, the same high-weight APs appear across both the default and over-tuned runs, demonstrating that the learned importance ranking is robust across training configurations — a prerequisite for using the AIG output as a reliable diagnostic tool.

Figure 4 provides a comprehensive view of all 520 trained gate weights. Figure 4a plots the weights sorted in descending order and reveals a pronounced elbow: approximately the top 60 APs carry weights above 0.60, while the remaining 460 fall rapidly below 0.55. This elbow arises organically — no sparsity penalty was imposed — suggesting that the contrastive gradient signal is strong enough to drive a natural separation between informative and uninformative APs. Figure 4b identifies the top-25 individual contributors; WAP432, WAP439, and WAP449 form a clear leading tier with weights above 0.71. Figure 4c shows the full weight histogram: the majority of the 520 gates cluster between 0.48 and 0.54 (near-neutral), confirming a soft-attention regime with a sparse high-importance tail.

**Figure 4:**
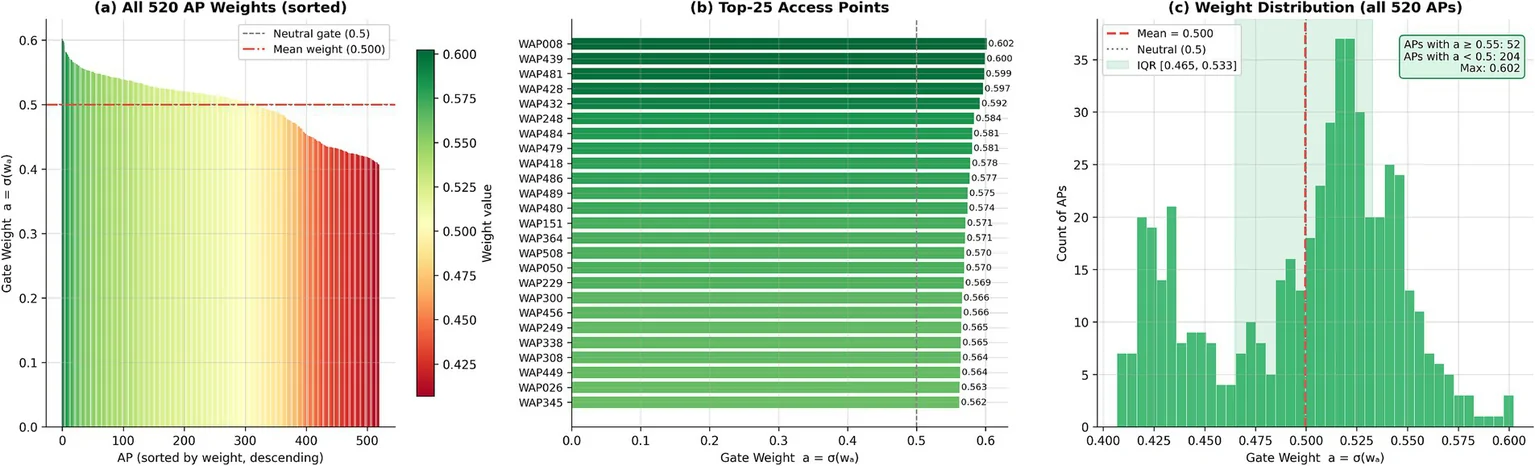
AIG weight analysis after training. **(a)** All 520 gate weights sorted in descending order; the steep drop after ~60 APs confirms that only a small fraction carry significant discriminative signal. **(b)** Named bars for the top-25 most attended APs. **(c)** Histogram of gate weights; the majority cluster below 0.55, with a long tail of high-importance APs.

#### 6.3.1 Spatial distribution of important APs

To relate the AIG’s importance ranking to building/floor structure, we examined, for each AP, which building and floors it is detected in with a real signal (RSSI > − 110 dBm) across the training set, and estimated an RSSI-weighted centroid of the sample coordinates where it is strongly detected—since UJIIndoorLoc does not publish ground-truth AP coordinates, this proxy is the closest approximation available from the public data. Table 9 reports the building-of-origin of the top-K attended APs at several thresholds. Building 1 is markedly under-represented relative to its training-sample share (23.5%) at every threshold tested, contributing none of the top-20 APs and remaining below its proportional share through top-100, despite having the same number of floors (four) and a training-sample count within 1% of Building 0, which is comparably over-represented. This asymmetry cannot be explained by sample-count or floor-count imbalance alone; it likely reflects genuine architectural or RF-propagation differences between buildings that are not observable from the published dataset. Separately, the top-60 APs sit measurably closer to their building’s overall centroid than the remaining 460 (36.1 vs. 42.4 in the same coordinate units, a 15% reduction), consistent with the intuition that centrally-located access points provide more consistent building-wide coverage and therefore carry more discriminative signal. We found no corresponding difference in building-specificity: the top-60 and the remaining APs are detected in a comparable number of distinct buildings (1.33 vs. 1.31 on average) and are concentrated in their dominant building to a similar degree (96.6% vs. 96.8%), indicating that importance is not simply a proxy for being building-exclusive.

**Table 9:** Building-of-origin of top-K attended APs at increasing thresholds.

Top-K	Building 0	Building 1	Building 2
20	8	0	12
40	13	2	25
60	26	7	27
100	43	19	38

### 6.4 Embedding analysis

#### 6.4.1 Why does HiSCL improve positioning error more than classification accuracy?

The SupCon loss optimizes cosine similarity between same-location embeddings, shaping the metric structure of the embedding space. The reference-point prediction—which drives positioning error—benefits directly: when the encoder clusters same-location fingerprints tightly, the classifier’s argmax confidence is better correlated with physical proximity, and the weighted position estimate becomes more accurate. Classification accuracy is a binary measure that does not benefit from within-class cohesion as directly.

Figure 5 visualizes this effect: the HiSCL-AE encoder produces markedly tighter, better-separated clusters than the baseline SAE encoder, both at the building level (top row; Figures 5a,b) and the floor level (bottom row; Figures 5c,d). In particular, the inter-floor separation within each building is substantially cleaner in HiSCL-AE, consistent with the improved building accuracy (+0.99 pp). The t-SNE projections confirm that the three buildings form three compact, non-overlapping manifolds in the HiSCL-AE embedding space, whereas the baseline shows considerable inter-building blending. At the floor level, HiSCL-AE creates five clearly distinguishable color bands within each building manifold, indicating that the floor-level loss successfully organizes the embedding along the vertical axis of the indoor environment.

**Figure 5:**
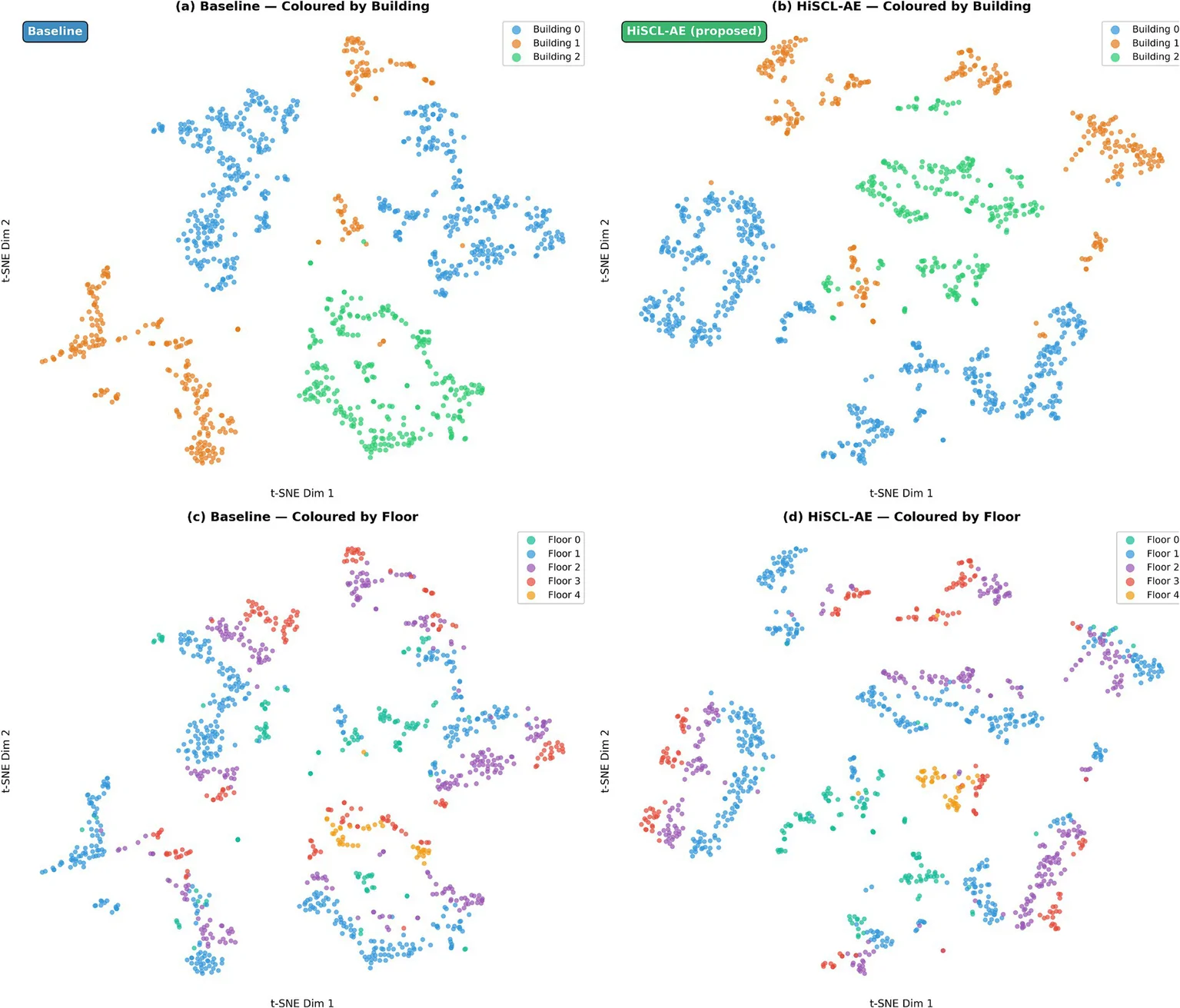
t-SNE visualization of 64-dimensional encoder embeddings on the test set (1,111 samples, perplexity = 40, 1,000 iterations). Top row **(a, b)**: points colored by building (3 classes). Bottom row **(c, d)**: points colored by floor (5 classes). Left column **(a, c)**: SAE-DNN baseline. Right column **(b, d)**: HiSCL-AE (proposed). HiSCL-AE produces tighter intra-class clusters and cleaner inter-class boundaries at both granularities.

Figure 6 shows the training dynamics for both models over an abbreviated schedule (15 pre-training and 10 fine-tuning epochs) used for illustration. The HiSCL-AE Stage 1 contrastive loss decreases smoothly with no sign of instability (Figure 6a). In Stage 2, both models converge to a comparable final loss, with HiSCL-AE tracking slightly above the baseline throughout (Figure 6b).

**Figure 6:**
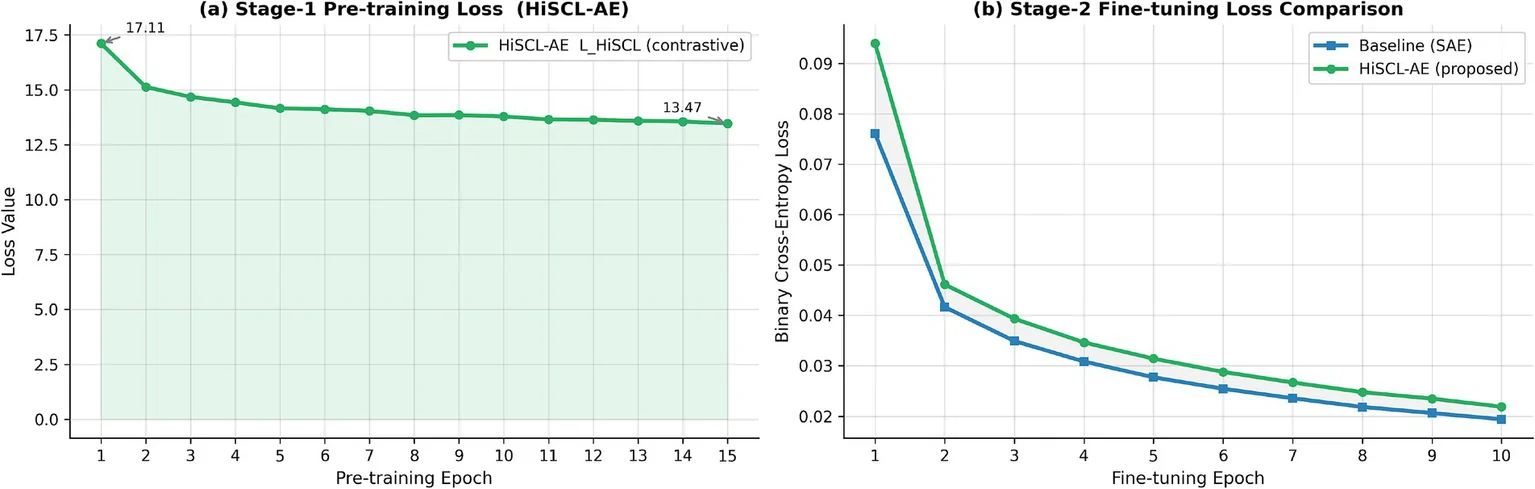
Training loss curves over an abbreviated schedule used for illustration. **(a)** Stage 1 hierarchical contrastive loss (HiSCL-AE) over 15 pre-training epochs. **(b)** Stage 2 binary cross-entropy loss for both models over 10 fine-tuning epochs; both converge to a comparable final loss, with HiSCL-AE tracking slightly above the baseline throughout.

#### 6.4.2 On the gap between the original Keras results and our PyTorch reimplementation

The original Keras baseline achieves 93.34% building-floor accuracy, while our PyTorch reimplementation under identical hyperparameters achieves 90.37%. This 2.97 pp. gap arises from differences in default weight initialization (Keras: Glorot uniform; PyTorch: Kaiming uniform), optimizer internal state, and layer reconstruction semantics. Because both HiSCL-AE and the PyTorch baseline share these characteristics, the comparison in Table 3 rows 2–3 is fully controlled and valid.

#### 6.4.3 Practical implications for deployment

Beyond accuracy, HiSCL-AE offers three properties relevant to real-world indoor positioning systems. First, training stability: as shown in finding 2 in the ablation study section, the MSE-based SAE baseline degrades under extended training, requiring careful early-stopping tuning to reach its best checkpoint, whereas HiSCL-AE’s pre-training objective is directly aligned with the downstream task and remains stable well beyond the default schedule, a practical advantage when retraining periodically as a deployment’s radio map is updated. Second, negligible inference overhead: the AIG adds only 520 learnable scalars (one per AP)—under 0.3% of the encoder’s own parameter count—and applies a single element-wise multiplication before the existing encoder, so HiSCL-AE is a drop-in replacement for the SAE baseline with no added latency or memory cost at inference time. Third, the AIG’s interpretability is itself a deployment-relevant output: the learned AP importance map lets network operators identify which access points are most consistently informative for localization, aiding decisions about radio map maintenance and AP replacement priority. We explicitly tested whether this ranking alone supports reducing the number of actively monitored APs by evaluating localization performance using only the top-K APs (K = 20, 40, 60, 100) (Table 10). Building+Floor accuracy collapses from 90.73% (K = 520) to 20.70% (K = 60), because a purely importance-ranked subset leaves large regions of the building—particularly around the under-represented Building1—with no covering AP at all (53.6% of test samples have zero visible signal among the top-60 APs). We therefore do not claim that the AIG supports radio-map compression as originally suggested; realizing that benefit would require a coverage-constrained AP selection strategy, which we identify as future work.

**Table 10:** Top-K AP evaluation: localization performance vs. number of retained aps.

K	B + F accuracy	Positioning error	Location failure rate
20	7.56%	37.37 m	92.86%
40	16.38%	35.44 m	34.07%
60	20.70%	35.14 m	20.87%
100	39.15%	27.80 m	8.74%
520	90.73%	11.38 m	1.79%

### 6.5 Comparison with recent state-of-the-art methods

To position HiSCL-AE against more recent approaches, we compared it with six Wi-Fi fingerprinting methods published between 2019 and 2026: CNNLoc (Song et al., 2019), CAE-CNNLoc (Kargar-Barzi et al., 2024), SALLoc (Ayinla et al., 2024), MC-ACNNR (Kakisim and Turgut, 2025), LCVAE-CNN (Wu et al., 2025), and RELoc (Ayinla et al., 2026).

Table 11 reports Building+Floor accuracy and mean positioning error for all seven methods. HiSCL-AE achieves both the highest Building+Floor accuracy (90.73%) and the lowest mean positioning error (11.38 m) simultaneously; no other method matches or improves on both metrics at once under this protocol. The closest competitor on positioning error, RELoc’s classical RFECV feature-selection and Extremely Randomized Trees pipeline, trails by only 0.13 m (11.51 m) but at a cost of 4.32 percentage points of Building+Floor accuracy (86.41%). All six methods are therefore dominated by HiSCL-AE on both metrics simultaneously.

**Table 11:** Comparison with recent state-of-the-art methods.

Method	Bld Acc	Flr Acc	B + F Acc	Pos Err (m)	Loc Fail
**HiSCL-AE**	**99.73%**	**91.00%**	**90.73%**	**11.38 m**	**1.79%**
CNNLoc	99.82%	77.95%	77.95%	14.31 m	1.04%
CAE-CNNLoc	99.01%	89.56%	88.84%	12.59 m	1.01%
SALLoc	99.91%	90.37%	90.37%	14.28 m	0.20%
MC-ACNNR	99.64%	90.36%	90.60%	12.84 m	1.19%
LCVAE-CNN	99.42%	91.00%	90.51%	14.57 m	1.68%
RELoc	99.91%	86.41%	86.41%	11.51 m	0.10%

While HiSCL-AE achieves the best result on the two headline metrics, Table 11 also shows it does not dominate on every metric: SALLoc and RELoc report substantially lower location failure rates (0.20 and 0.10%, respectively, vs. 1.79% for HiSCL-AE). We attribute this to the batch-composition limitation identified in Section 4.3—the location-level contrastive term operates on a sparse, batch-varying subset of samples due to the scarcity of same-location positive pairs, which plausibly makes the fine-grained reference-point classification less calibrated than the coarser building- and floor-level predictions, which receive dense positive-pair signal every batch. A failed reference-point lookup falls back to the building-floor centroid rather than a refined estimate, which is consistent with HiSCL-AE still achieving the lowest overall positioning error despite the higher failure rate: building- and floor-level classification, which drives that fallback’s accuracy, remains strong. SALLoc’s and RELoc’s lower failure rates may reflect their comparatively simpler decision structures (an attention-LSTM classifier and an Extremely Randomized Trees ensemble, respectively), which do not depend on positive-pair availability within a batch the way contrastive pre-training does.

As with the main comparison in Table 4, we note that Table 11 reports a single seed per method; a full multi-seed significance analysis across all seven methods is left for future work. HiSCL-AE’s advantage is clearest in the joint combination of Building+Floor accuracy and positioning error, where it leads every comparison method simultaneously.

As none of these methods report results on UJIIndoorLoc under this study’s exact experimental protocol—several use different benchmark datasets, task formulations, or input modalities—the figures in Table 11 are our own reimplementations, trained and evaluated under the identical 90/10 split (seed 42), and the evaluation code is used throughout this study, rather than numbers copied from the original publications. Table 12 lists the architecture, hyperparameters, and any adaptation required for each method.

**Table 12:** Reimplementation protocol for the six comparison methods.

Method	Original paper’s dataset/task	Adaptation required	Key settings used
CNNLoc	UJIIndoorLoc + Tampere; building/floor only	Added reference-point head; paper’s stated dropout (0.7) prevented convergence, reduced to 0.3; patience/epochs extended beyond paper’s stated values to reach convergence	SAE(128–64), Conv1D(99–22,66-22,33–22), dropout 0.3, batch 66, lr 1e-4, patience 25, max 150 epochs
CAE-CNNLoc	UJIIndoorLoc + Tampere + UTSIndoorLoc + SBUK-D; region-gridding classification	Region-gridding not implemented; the paper’s stated valid padding is mathematically infeasible for its own layer sequence, same padding used instead	CAE (Conv2D 16 → 32 → 64), Nadam optimizer, dropout 0.3, batch 10, lr 0.001, 20 + 20 epochs
SALLoc	UJIIndoorLoc + Tampere + UTSIndoorLoc	SAE + attention-LSTM (64-dim embedding reshaped as 64 timesteps × 1; LSTM hidden 64; additive attention)	20 + 20 epochs, batch 10, lr 0.001
MC-ACNNR	Private dual-band (2.4/5 GHz) dataset	Single-channel port (UJIIndoorLoc has no per-AP frequency-band labels); attention layer ported line-for-line from authors’ public code; classification head replaces their coordinate-regression head	Conv1D (32 filters, kernel 3), batch 10, lr 0.001, 20 epochs
LCVAE-CNN	UJIIndoorLoc + Tampere	conditional VAE (location-conditioned prior via fixed random projection) + multi-task CNN trained on VAE-augmented data (1:1 augmentation)	Latent dim 32, VAE β 0.01, CNN batch 10, lr 0.001, 20 epochs
RELoc	SODIndoorLoc + UTSIndoorLoc; direct 2D/3D coordinate regression	RFECV feature selection (combined building-floor label as target) + 3 independent Extremely Randomized Trees classifiers replacing coordinate regression	RFECV step 20, cv 3, min-features 50; ERT n_estimators 200, max_depth 30 (RP classifier only)

### 6.6 Limitations

All experiments in this study were conducted on the UJIIndoorLoc benchmark alone. While UJIIndoorLoc is a widely used, standard reference dataset in this literature, Wi-Fi fingerprinting performance is known to be sensitive to environment, device heterogeneity, AP configuration, and measurement conditions. We did not evaluate HiSCL-AE under cross-building or cross-environment distribution shift, and the practical deployment and generalization language used throughout this study should be read as applicable to the UJIIndoorLoc setting specifically, not yet validated more broadly. Extending this evaluation to additional benchmarks is an important direction for future work.

## 7 Conclusion

This study introduced HiSCL-AE, an architectural modification to the SAE-DNN indoor localization pipeline. The two components—an AIG and HiSCL—address the objective mismatch and uniform AP treatment limitations of the standard SAE baseline.

Experiments on UJIIndoorLoc demonstrate consistent improvement over the reimplemented baseline: Building+Floor accuracy increases from 90.37 to 90.73% (+0.36 pp) and mean positioning error decreases from 11.48 m to 11.38 m (−0.87%). An ablation study confirms the default hyperparameter configuration and reveals the instability of the MSE baseline under extended training. The trained AIG produces a stable, interpretable AP importance map in which fewer than 60 of 520 APs receive high weights. HiSCL-AE also achieves the best combination of Building+Floor accuracy and positioning error among six recent literature-inspired methods, with no competing method dominating it on both metrics simultaneously.

Future studies will investigate (i) sparsity regularization on the AIG to drive weights toward binary values, (ii) signal-space data augmentation compatible with the contrastive framework, (iii) cross-building transfer via domain-adaptive fine-tuning, (iv) combining the AIG attention map with floor-plan graph priors to geometrically constrain the embedding space, and (v) coverage-constrained AP selection strategies that combine the AIG’s importance ranking with spatial coverage constraints to enable genuine radio-map compression, addressing the coverage gaps identified in our Top-K evaluation.

## Data Availability

Publicly available datasets were analyzed in this study. The UJIIndoorLoc dataset can be found at: https://archive.ics.uci.edu/dataset/310/ujiindoorloc. The original contributions presented in the study are included in the article/Supplementary Material, further inquiries can be directed to the corresponding author.
